# Fanconi anemia genes in lung adenocarcinoma- a pathway-wide study on cancer susceptibility

**DOI:** 10.1186/s12929-016-0240-9

**Published:** 2016-02-03

**Authors:** Shi-Yi Yang, Chia-Ni Hsiung, Yao-Jen Li, Gee-Chen Chang, Ying-Huang Tsai, Kuan-Yu Chen, Ming-Shyan Huang, Wu-Chou Su, Yuh-Min Chen, Chao A. Hsiung, Pan-Chyr Yang, Chien-Jen Chen, Pei-Ei Wu, Jyh-Cherng Yu, Chen-Yang Shen, Huan-Ming Hsu

**Affiliations:** Genomics Research Center, Taipei, Taiwan; Graduate Institute of Epidemiology, College of Public Health, National Taiwan University, Taipei, Taiwan; Institute of Biomedical Sciences, Taipei, Taiwan; Division of Chest Medicine, Department of Internal Medicine, Taichung Veterans General Hospital, Taichung, Taiwan; Department of Pulmonary and Critical Care, Chang Gung Memorial Hospital, Lincou, Taiwan; Department of Internal Medicine, National Taiwan University Hospital and National Taiwan University College of Medicine, Taipei, Taiwan; Department of Internal Medicine, Kaohsiung Medical University Hospital, Kaohsiung, Taiwan; Department of Internal Medicine, National Cheng Kung University Hospital and College of Medicine, Tainan, Taiwan; Chest Department, Taipei Veterans General Hospital, Taipei, Taiwan; Division of Biostatistics and Bioinformatics, Institute of Population Health Sciences, National Health Research Institutes, Zhunan, Taiwan; Taiwan Biobank, Taipei, Taiwan; Life Science Library, Academia Sinica, Taipei, Taiwan; Graduate Institute of Environmental Science, China Medical University, Taichung, Taiwan; Division of General Surgery, Department of Surgery, Tri-Service General Hospital, National Defense Medical Center, Taipei, Taiwan

**Keywords:** Lung adenocarcinoma, DNA repair, Fanconi anemia, Genetic susceptibility, Polymorphism

## Abstract

**Background:**

Carcinogens in cigarette smoke can induce the formation of DNA-DNA cross-links, which are repaired by the Fanconi anemia (FA) pathway, and it is tempting to speculate that this pathway is involved in lung tumorigenesis. This study is to determine whether genetic polymorphism of the FA genes is associated with an elevated risk of lung adenocarcinoma, and whether the association between genotypes and risk is modified by exposure to cigarette smoke.

**Methods:**

This case–control study genotyped 53 single-nucleotide polymorphisms (SNPs) in FA genes in 709 patients (354 males and 355 females) with lung adenocarcinoma and in 726 cancer-free individuals (339 males and 387 females). Genotypic frequencies of SNPs were compared between cases and controls to identify important FA genes associated with cancer susceptibility. Joint effects in determining cancer risk contributed by genes and smoking-related risk factors and by multiple genes involved in different FA subpathways were evaluated by multivariate regression analysis and stratified analysis. All analyses were performed on males and females separately, and the comparison of results was considered a way of examining the validity of study findings.

**Results:**

Lung adenocarcinomas in both male and female patients were associated with (a) genotypic polymorphisms of FANCC and FANCD1; (b) a combined effect of harboring a higher number of high-risk genotypes and smoking/passive smoking; (c) specific interactions of multiple genes, proteins encoded by which have been known to work jointly within the FA pathway.

**Conclusions:**

Genetic polymorphism of the FA genes is associated with inter-individual susceptibility to lung adenocarcinoma.

**Electronic supplementary material:**

The online version of this article (doi:10.1186/s12929-016-0240-9) contains supplementary material, which is available to authorized users.

## Background

Cigarette smoking, the most important risk factor causally linked to lung tumorigenesis, is associated with a four-fold increase in lung cancer risk. Although 80 % of lung cancers can be attributed to such exposure [1, 2], only 15 % of smokers develop lung cancer [2, 3], strongly suggesting the importance of inter-individual genetic susceptibility in modulating the risk of developing disease. The carcinogenic effect of smoking is caused by attack by tobacco metabolites on DNA to form DNA adducts.

Cytochrome P450 enzymes are responsible for the activation and detoxification of these metabolites [4] and it has long been suggested and indeed shown that low-penetrance polymorphic alleles of genes encoding these enzymes are important in determining genetic susceptibility to lung cancer [4–6]. In addition to the neutralization of carcinogens by these enzymes before they can damage DNA, cells have developed overlapping pathways to repair this type of damage, thus protecting genomic integrity [7]. An association between lung cancer risk and genetic polymorphisms of DNA repair genes has been demonstrated [8–10]. The genes involved include those involved in the nucleotide excision repair pathway, which removes bulky adducts, and in the base excision repair pathway, which deals with small lesions caused by alkylating agents or reactive oxidative species in cigarette smoke. Some important chemicals in cigarette, e.g., 1,2,3,4-diepoxybutane (DEB) [11, 12], induce formation of DNA-DNA adducts by sequentially alkylating two nucleobases within the DNA double helix. If left unrepaired, this cross-linking would lead to a variety of genotoxic effects, including point mutations, large deletions, and chromosomal aberrations commonly seen in lung cancer [13]. The Fanconi anemia (FA) pathway regulates the repair of DNA crosslinking damage [14–16], and it is tempting to speculate on a possible lung tumorigenic role of this pathway. To test this hypothesis, this case–control study examined whether genetic polymorphism of FA pathway genes is associated with an elevated risk of lung adenocarcinoma and whether the association between genotypes and risk is modified by exposure to cigarette smoke.

To date, eighteen FA pathway genes have been identified, germline mutation of which results in a common FA phenotype, a rare genetic disorder characterized by aplastic anemia, cancer/leukemia susceptibility, and cellular hypersensitivity to DNA crosslinking agents [14, 17, 18]. The proteins encoded by individual FA genes form three functional complexes (the nuclear core complex, ubiquitinated complex, and repair complex), which act cooperatively and sequentially to repair DNA crosslinking damage [14, 15, 19–26]. BRCA2 (FANCD1), PALB2 (FANCN), and BRIP1 (FANCJ) interact with BRCA1 for DNA crosslinking repair [27, 28]. Recently, the presence of biallelic *BRCA1* mutations in some patients with multiple congenital anomalies consistent with a FA-like disorder [28]. Based on these molecular interactions operating within and between the individual steps of the FA pathway, our comprehensive examination of the association between genetic variants of all FA genes and tumorigenesis of lung adenocarcinoma provides a unique opportunity for exploring whether joint effects of genes coding for proteins in the different complexes of the FA pathway determine cancer risk.

## Methods

### Study subjects

We enrolled subjects who were participating in the Genetic Epidemiological Study of Lung Adenocarcinoma (GELAC) in Taiwan, a molecular epidemiological study on genetic susceptibility markers for lung cancer [29, 30]. Because of the homogenous genetic background of the Taiwanese population, its use reduces the chance of false positives due to population stratification [31]. The study aim of the GELAC is mainly focused on patients with lung adenocarcinoma, as adenocarcinoma is the most common histological type of lung cancer in Chinese women in Taiwan [32]. Lung cancer patients, aged 18 years or older, with histological or cytological diagnoses were recruited from 6 tertiary medical centers in Taiwan. The study included 354 male lung adenocarcinoma patients and 355 female lung adenocarcinoma patients, recruited between September 2002 and December 2005. Patients with a history of other cancers were excluded from this study. These patients accounted for almost all (>95 %) subjects with lung cancer attending our lung cancer clinics during this period, the other patients being excluded because of a lack of suitable blood specimens. No significant differences in risk factors of lung cancer were found between the included and excluded subjects.

The control subjects were cancer-free individuals (339 males and 387 females) randomly selected from the health examination clinics of the same hospitals during the same period of case recruitment [29, 30]. Individuals with a history of cancer or showing any evidence of suspicious precancerous lesions were excluded as controls. Eighty-seven percent of eligible controls agreed to take part. The study was performed after approval by the institutional review board of each participating institute (National Health Research Institutes).

### Questionnaire and specimen collection

At recruitment, trained research nurses were assigned to obtain informed consent for the collection of a blood sample and to administer a structured questionnaire. The questionnaire collected information about demographic characteristics, lifestyle factors (such as number of cigarettes smoked), medical history, and family history of cancer. In terms of smoking status, a person who was currently smoking at least one cigarette a day and had been doing so for more than 6 months was regarded as a current smoker. The validity of the questionnaire has been addressed and confirmed in our previous studies [29, 30].

### SNP selection and genotyping

**Fig. 1:**
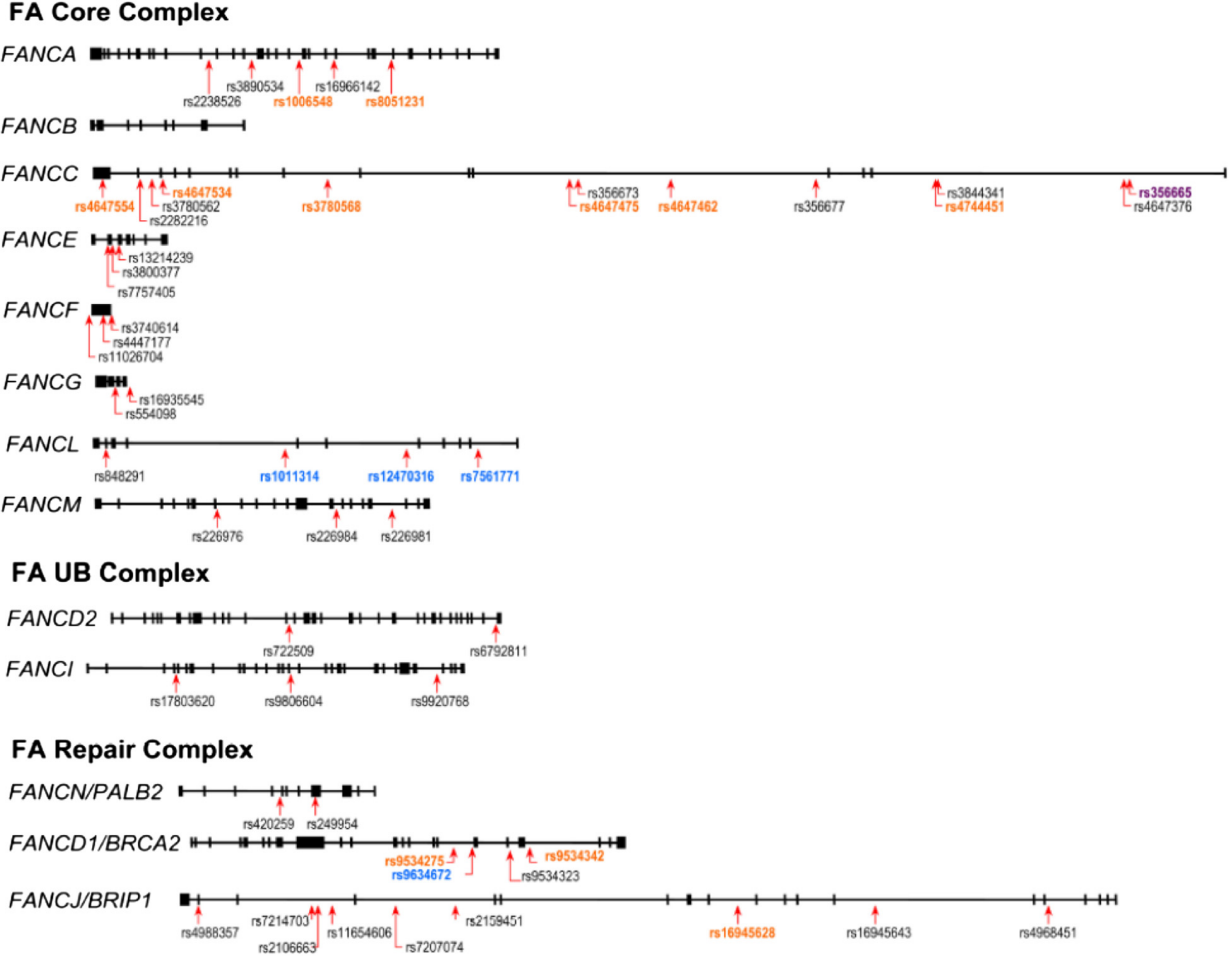
Schematic diagram of the Fanconi anemia (FA) genes and locations of the genotyped single-nucleotide polymorphism (SNPs) in individual FA genes. The length of the line representing each FA gene is proportional to the size (i.e., base pair) of the gene. *Black boxes,* exons; *arrows,* positions of the SNPs genotyped and analyzed in this study. The SNPs marked in orange are those significantly associated with lung adenocarcinoma in females, those marked in light blue are significantly associated with lung adenocarcinoma in males, and that marked in purple (i.e., rs356665 in *FANCC)* is significantly associated with lung adenocarcinoma in both males and females

Genomic DNA was extracted from blood specimens from all subjects using the conventional salting-out procedure. In this study, in addition to the size of the genes and a >5 % minor allele frequency of SNPs, the haplotype block structure of individual FA genes was used to select SNPs. All SNPs were chosen based on the information for the Chinese population in the HapMap database (Additional file 1: Figure S1) [33]. In theory, for SNPs that are in strong linkage (i.e., linkage disequilibrium, LD), a limited number of tag SNPs is enough to reflect the polymorphic status of the gene. We followed this principle to choose SNPs, ensuring that at least one SNP was selected for each haplotype block of each FA gene, and a total of 65 SNPs was chosen. *FANCB* was not included in this study because it is located on the X chromosome, which suggests than any association would be less in male lung cancer patients than in females, and, more importantly, no SNP in *FANCB* has yet been found in the Chinese population. A genotyping platform based on the 5′ nuclease allelic discrimination TaqMan assay in a 96-well format on an ABI Prism 7900HT Sequence Detection System (Applied Biosystems) was used for genotyping. In an initial screening of 96 cases and 96 controls, all 65 SNPs were genotyped. Of these, 12 were not observed or were infrequent (frequency of the minor allele < 0.01), so these SNPs were not genotyped in the rest of the samples. The remaining 53 SNPs (Fig. 1) were genotyped in all cases and controls. The representativeness of these selected SNPs is supported by the results of genotyping in our controls, showing that, in individual genes, almost all blocks of a gene were represented by at least one SNP (Additional file 2: Figure S2). To ensure that the observed polymorphisms were specific and not the result of experimental variation, 10 % of the samples were randomly selected and run in duplicate, with complete concordance of results.

### Data analysis

The following sequential statistical analyses [34, 35] were used.

(i) Univariate and multivariate analyses were used to determine risk factors and to establish background risk profiles for lung adenocarcinoma in this series of study subjects. Smoking-related risk factors, i.e., a history of cigarette smoking or passive smoking, were used as indices to estimate the effect of smoking and the degree of carcinogen exposure in the subsequent analysis.
(ii) To ensure that the controls used were representative of the general population and to exclude the possibility of genotyping error, deviation of the genotype frequencies of each SNP in the control subjects from that expected under Hardy-Weinberg equilibrium was assessed using the goodness-of-fit test. The degree of LD between markers was indicated using Lewontin’s D’ value.
(iii) Differences in genotypic frequencies of individual SNPs between cases and controls were tested using the chi-squared and Fisher’s exact tests. Multiple logistic regression analysis with simultaneous consideration of known risk factors of lung adenocarcinoma was performed and the adjusted odds ratios (aORs) for the associations estimated.
(iv) Because we were especially interested in the relationship between the FA genes and lung adenocarcinoma risk within categories of smoking-related risk factors representing different degrees of smoke exposure, we calculated the risk of lung adenocarcinoma associated with the combination of the number of putative high-risk genotypes of FA genes and a given smoking-related risk factor. Using ß estimates from the logistic regression model, in which we used a set of dummy variables representing different combinations of genes (*i.e.*, number of putative high-risk genotypes) and a history of smoke exposure, we assessed the relative excess risk from harboring higher numbers of putative high-risk genotype within risk factor strata (the joint method described in refs. [35] and [36].
(v) Based on the known interactions of the proteins in the FA pathway (Additional file 1: Figure S1), we applied a combination of the joint and stratified methods [35, 36] to determine whether specific known molecular interactions among FA proteins are associated with lung adenocarcinoma susceptibility. We therefore first stratified the subjects on the basis of the genotypic status of the most functionally important FA gene, as determined in previous molecular and cellular studies, then a joint effect of FA genes on increased lung adenocarcinoma risk was explored using conventional logistic regression, a test evaluating whether a statistically significant increase in risk is observed with specific combinations of putative high-risk genotypes in these genes (measured by the ß estimates from this regression model). An alternative method would be to use the p value for interaction to examine whether the “interaction” of two/multiple genes is associated with cancer risk. However, this p value is based on an assumption of a multiplicative effect, and, though statistically justified, this model may not reflect the combined effects of genes coding for proteins in the FA pathway.

Though a gender effect can be adjusted in data analysis, we deliberately performed all analyses on males and females separately. Because they all had adenocarcinoma of the lung sharing common etiologies, if the FA pathway plays a role in lung tumorigenesis, similar results may be expected for the male and female lung adenocarcinoma patients.

Accordingly, the comparison of results between genders can be considered a way of testing the validity of our findings.

## Results

The risk profile of this series of study subjects was similar to that reported in our previous studies [29, 30], and, using multivariate logistic regression analysis considering all known risk factors of lung cancer, smoking-related risk factors were found to play a dominant role in determining lung adenocarcinoma risk. As shown in Table 1, in males, almost 72 % of cancer patients had a history of cigarette smoking of more than six months as compared to 49 % in controls, resulting in a significant aOR of 1.82 [95 % confidence interval (95 % CI), 1.28–2.56]. In addition, 85.3 % of male patients reported having been frequently exposed to passive smoking compared to 64.9 % of controls, and this was associated with a higher odds ratio of 2.68 (95 % CI, 1.79–4.02). In females, as expected, due to the fact that cigarette smoking is relative uncommon in Asian women, this putative risk factor had no significant effect on risk. However, as in males, a history of passive smoking was reported very frequently in females (74.9 % of cases and 57.6 % of controls) and was the most important risk factor, associated with a two-fold increase (aOR, 2.13; 95 % CI, 1.53–2.95) in risk.

**Table 1:** Distribution of risk factors of lung adenocarcinoma and adjusted odds ratios in relation to risk of lung adenocarcinoma in males and females in Taiwan

	Male			Female		
Risk factor	No. cases (%)	No. controls (%)	aOR (95 % CI)^a^	No. cases (%)	No. controls (%)	aOR (95 % CI)^a^
Cigarette smoking						
> = 6 months	255(72.0)	166(49.0)	1.82(1.28–2.56)	17(4.8)	13(3.4)	1.18(0.54–2.54)
Passive smoking						
Yes	302(85.3)	220(64.9)	2.68(1.79–4.02)	266(74.9)	223(57.6)	2.13(1.53–2.95)
Smoke exposure during cooking						
Yes	NA	NA	NA	319(89.9)	339(87.6)	1.05(0.66–1.68)
Using motor cycle for transport						
> = 6 months	239(67.5)	157(46.3)	2.00(1.42–2.81)	165(46.5)	164(42.4)	1.34(0.95–1.92)

^a^aOR, adjusted odds ratio; 95 % CI, 95 % confidence interval. These were estimated in a multivariate logistic regression model, containing age, years of schooling of study participants, and the risk factors listed in this Table

All 53 SNPs were in Hardy-Weinberg equilibrium in the controls. To obtain initial information about a possible association between lung adenocarcinoma and SNPs of the FA genes, the genotype distributions of these SNPs were compared between cases and controls in males and females separately. We estimated the risk (aOR) associated with harboring both the homozygous variant genotype and one additional at-risk allele, and 16 SNPs were found to show a statistically significant difference (Fig. 1). Notably, most of these were clustered within a given gene, multiple significant SNPs being found in *FANCA, FANCC,* and *FANCD1* in female patients and in *FANCL* in male patients. This suggests that the association detected may not be due solely to chance. The reliability of these associations was supported by the finding that SNPs in *FANCC* and *FANCD1* were associated with lung adenocarcinoma in both males and females, consistent with the fact that the lung cancers were all adenocarcinomas and would share a similar molecular etiology in both sexes, such as susceptibility genes. Interestingly, one SNP, *rs356665,* located at the 3′-end of *FANCC,* was found to be significant in both male and female lung adenocarcinoma patients. However, in females, other SNPs in *FANCC* also showed a significant association, while, in males, *rs356665* was the only significant *FANCC* SNP in determining risk. To explain this, we checked the LD among SNPs, and found that *rs356665* was located in a different haplotype block compared to other SNPs of *FANCC* in both males and females (Additional file 3: Figure S3). One possibility is that FANCC has multiple functional domains, and that, in males, only the domain encoded by the 3′-end sequence is important, while all domains are associated with susceptibility in female lung adenocarcinoma patients.

To comprehensively assess the individual contribution of different FA genes in the association with lung adenocarcinoma development, we performed a logistic regression analysis considering the effects of both environmental risk factors and individual genes simultaneously. As shown in Table 2, consistent with the findings of the single SNP/gene analysis, the high-risk *FANCC* and *FANCD1* genotypes were strongly associated with lung adenocarcinoma risk in both males and females. In addition, polymorphisms of *FANCL* or *FANCJ* were associated with a relatively minor, but still significant, risk in male or female lung adenocarcinomas, respectively.

**Table 2:** Unconditional logistic regression analysis of genotype polymorphisms of FA genes and multiple risk factors for lung adenocarcinoma development

Risk factor	Multivariate aOR^a^	95 % CI^a^
Male lung cancer patients		
FA gene		
*FANCC(rs356665)*		
*AA vs. AG,GG*	2.06	1.13–3.72
*FANCD1(rs9634672)*		
*TT vs. GT,GG*	2.24	1.31–3.85
*FANCL(rs12470316)*		
*TT vs. CT, CC*	1.78	1.02–3.13
Cigarette smoking		
*Yes vs. No*	2.29	1.80–4.46
Passive smoking		
*Yes vs. No*	2.83	1.79–4.46
Female lung cancer patients		
FA gene		
*FANCC(rs4744451)*		
*GG vs. GT,TT*	1.67	1.01–2.75
*FANCD1(rs9534275)*		
*AA vs. AC,CC*	1.62	1.20–2.32
*FANCJ/BRIP1/BACH1(rs16945628)*		
*CC vs. CT, TT*	1.44	1.02–2.01
Passive smoking		
*Yes vs. No*	2.14	1.50–3.03

^a^aOR, adjusted odds ratio; 95 % CI, 95 % confidence interval. These were estimated in a multivariate logistic regression model, containing age and years of schooling of study participants

**Fig. 2:**
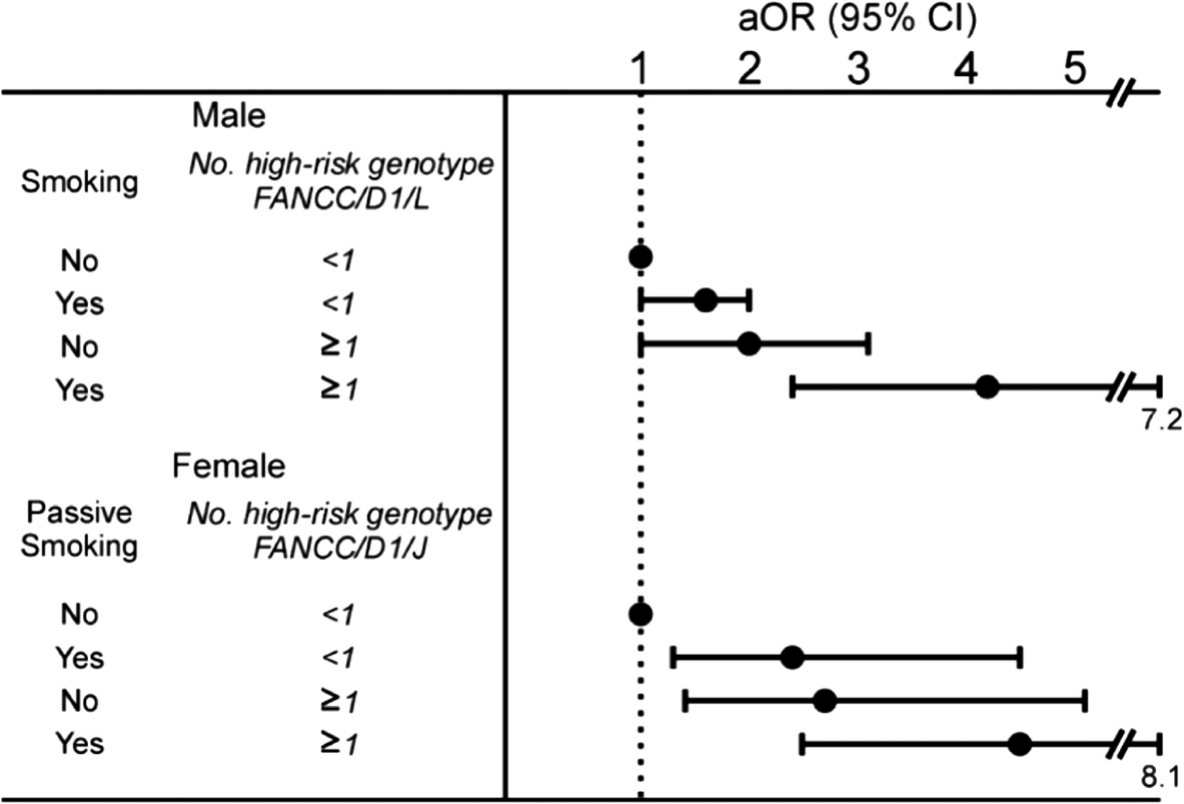
Risk (adjusted odds ratio, aOR, 95 % confidence interval, 95 % CI) of lung adenocarcinoma in males and females associated with a joint effect of harboring a higher number of putative at-risk genotypes of Fanconi anemia (FA) genes and smoking or passive smoking

**Fig. 3:**
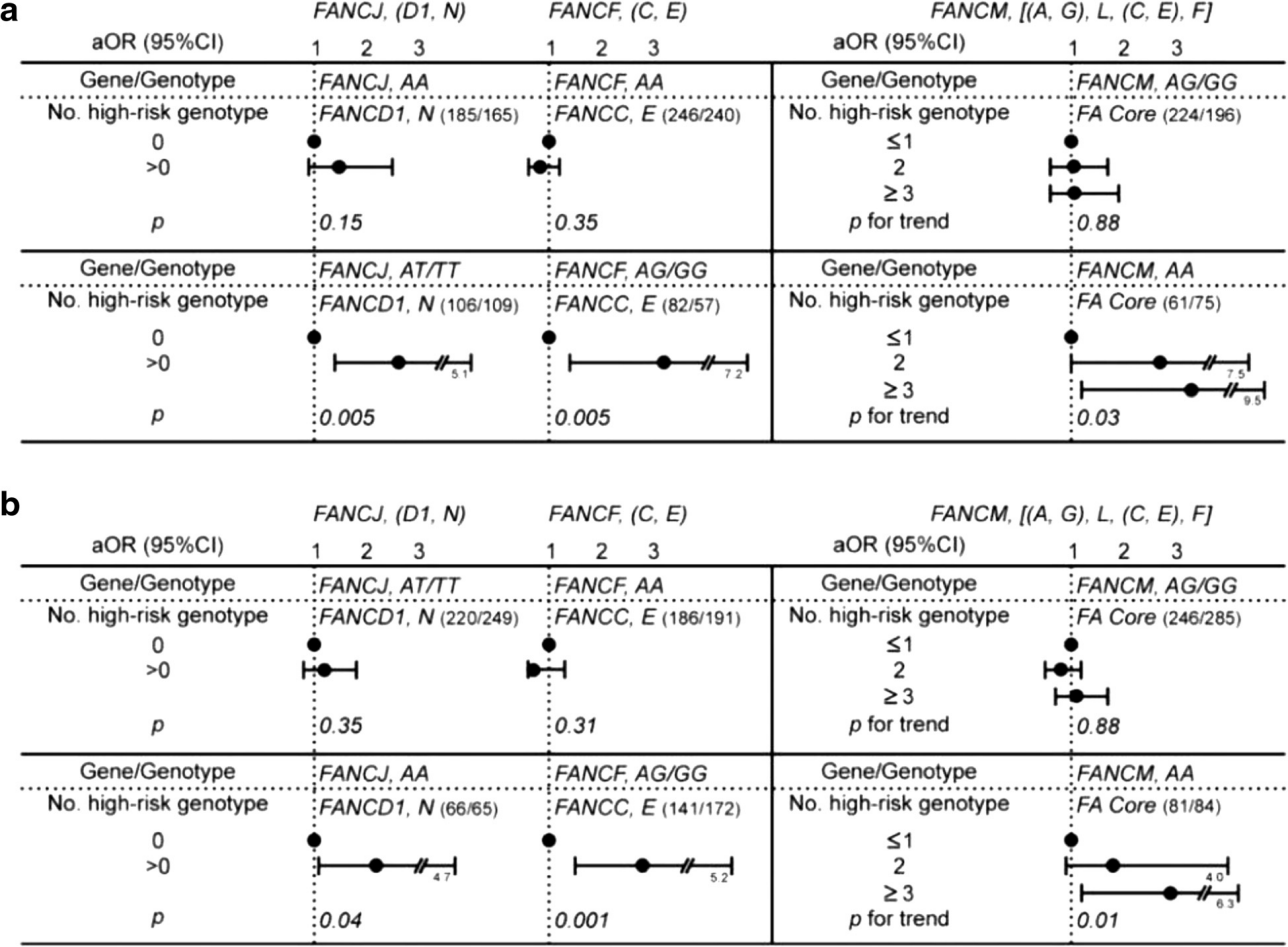
Interaction between functionally-related Fanconi anemia (FA) genes in determining lung adenocarcinoma risk in males (**a**) and females (**b**), assessed by stratified analysis. Lung adenocarcinoma risk associated with the number of high-risk genotypes of FA genes in males or females stratified by genotypes of specific functionally-interacting FA genes. See the Results section for details of the putative mechanisms involved in these interactions. The numbers in parenthesis in each stratum are the number of cases/number of controls in this stratum

If these FA genes were associated with lung adenocarcinoma development via the hypothesized mechanism involving the repair of cross-linking damage caused by smoke, the relationship between cancer risk and susceptibility genotypes would be expected to be more significant in the subset of individuals with a history of cigarette smoking or passive smoking. We therefore investigated the potential importance of smoke exposure in conjunction with the three susceptibility genotypes identified in Table 2. Our suggestion was supported by the findings shown in Fig. 3, which showed that cigarette smoke may modify the association between the number of high-risk genotypes of FA genes and increased cancer risk. A more significant association of an increased cancer risk contributed by a higher number of high-risk genotypes was seen in males with a history of cigarette smoking and in females with a history of passive smoking (Fig. 2). In contrast, among individuals without a history of smoking or passive smoking, the lung adenocarcinoma risk conferred by a high number of high-risk genotypes was less.

No evidence was found for a role of genes of the FA UB complex (i.e., *FANCD2* and *FANCI*) in lung adenocarcinoma susceptibility. This was unexpected, because the corresponding proteins are known to play a central role in switching the FA DNA damage response and in linking the FA core complex with the FA repair complex to repair cross-linking damage [14, 20, 37, 38]. One possible explanation is that, because the genes coding for the UB complex are crucial in the FA pathway, any severe defects in them would result in genomic instability and trigger cell death by cell cycle checkpoint surveillance [39]. Thus, for these high-penetrant FA genes, only subtle defects arising from low-penetrance (risk) alleles would escape checkpoint surveillance and allow accumulation of the unrepaired DNA damage required for tumor formation. As a result, only a combination of functionally-related low-penetrance alleles would be found to have a significant effect on cancer risk [40]. To address this possibility, we generated a two (disease status, *i.e.*, case and control)-by-four (genotype status of both *FANCD2* and *FANCI*) table, in which individuals with putative low-risk genotypes of both *FANCD2* and *FANCI* served as the reference group. When arranged in this way, the data showed that the aOR for the putative high-risk genotype of *FANCD2* or *FANCI* alone was not significant, whereas the aOR for both together was significant in both males and females (Table 3), a result consistent with a joint effect of two FA UB complex subunits on lung adenocarcinoma risk.

**Table 3:** Lung adenocarcinoma risk associated with the number of high-risk genotypes of *FANCD2* and *FANCI* in male and female lung adenocarcinoma patients-Joint effect of component genes in the FA UB complex

Genotype of		No. cases (%)	No. controls (%)	aOR (95 % CI)^a^	*P* for trend
*FANCD2* ^b^	*FANCI* ^b^				
Male lung adenocarcinoma patients					
*AA*	*CC*	82(27.1)	94(33.6)	1.00(ref.)	0.03
*AA*	*CC, CT*	104(34.3)	94(33.6)	1.27(0.94–1.93)	
*AG,GG*	*CC*	50(16.5)	44(15.7)		
*AG,GG*	*CC,CT*	67(22.1)	48(17.1)	1.83(1.08–3.13)	
Female lung adenocarcinoma patients					
*AA*	*CC*	42(14.1)	63(18.9)	1.00(ref.)	0.02
*AA*	*CC,CT*	65(21.9)	57(17.1)	1.52(0.95–2.42)	
*AG,GG*	*CC*	87(29.3)	114(34.1)		
*AG,GG*	*CC,CT*	103(34.7)	100(29.9)	1.81(1.10–2.99)	

^a^aOR, adjusted odds ratio; 95 %CI, 95 % confidence interval. These were estimated in a multivariate logistic regression model, containing age, years of schooling of study participants, and the smoking-related risk factors (i.e., cigarette smoking and passive smoking) identified in Table 2

^b^The SNPs used in the analysis were rs6792811 (*FANCD2*) and rs9806604(*FANCI*) in males and rs6792811(*FANCD2*) and rs9920768(*FANCI*) in females

Recent studies have shown that individual FA proteins act jointly within different FA complexes (Additional file 1: Figure S1), and there are interactions between the different FA proteins within an individual FA complex, sequentially affecting specific functions of DNA repair [14, 19, 20]. Thus, it was intriguing to determine whether these interactions between FA proteins may be associated with lung adenocarcinoma development. To this end, we performed a stratified analysis [34]. If these FA genes were associated with cancer via the hypothesized mechanism involving the molecular interaction of component proteins within the FA complex, the relationship between cancer risk and susceptibility genotypes would be expected to differ in cases/controls harboring different genotypes of the most functionally critical FA genes in specific complexes. We therefore focused on the interactions within (a) the FA repair complex, (b) the FANCF/C/E complex, and (c) the FA core complex, in which genotypes of *FANCJ, FANCF,* and *FANCM* were used as respective stratification markers. The rationale underlying this strategy is based on the evidence that: (a) within the FA repair complex, FANCD1 and FANCN have been purified as a sub-complex different from that containing FANCJ, and each of these subcomplexes interacts with different repair proteins and is responsible for different steps in repairing cross-linking damage [14, 41]; (b) FANCF acts as an adaptor protein that plays a key role in the assembly of FANCC/E with the other FA proteins in the FA core complex [14, 42, 43]; (c) FANCM has ATP-dependent DNA translocase activity, allowing it to act as an engine to translocate the FA core complex along DNA to the damage site [14, 44, 45]. The results shown in Fig. 3 are consistent, in both males (A) and females (B), with combined effects of component FA genes in determining cancer risk. Using the interaction between *FANCM* and other component genes in the FA complex as an example (right side of Fig. 3), a significant increasing trend (*P* < 0.05) in lung adenocarcinoma risk associated with a higher number of high-risk genotypes of the component genes within the FA core complex was only seen in individuals with the putative high-risk genotype of *FANCM*. Similar patterns were observed in the other two interactions (left side of Fig. 3), a significant increasing trend (*P* < 0.05) associated with *FANCD1/N* or *FANCC/E* only being found in the strata of specific genotypes of *FANCJ* or *FANCF*, respectively. The possibility of a difference in statistical power in the detection of cancer risk due to different sample sizes in the subsets of study subjects can be excluded, as there were actually fewer study subjects in the strata showing significant *P* values. It is notable that both female and male lung adenocarcinoma patients gave the same results.

## Discussion

Based on a multigenic model and known molecular interactions between proteins involved in DNA repair mechanism and using an epidemiologic approach, the present study examined the contribution to lung tumorigenesis of the genes encoding the proteins in the FA pathway, the key mechanism involved in repairing DNA cross-linking damage. This study addressed not only the lung tumorigenic risk associated with the FA genes, but also whether there is a combined effect of these genes and cigarette smoke in relation to lung adenocarcinoma development and whether a joint effect of component FA genes within different FA complexes is important in determining cancer risk. Support for the role of genetic polymorphism of the FA genes associated with inter-individual susceptibility to lung adenocarcinoma came from the combination of three lines of evidence; Lung adenocarcinomas in both male and female patients were consistently associated with (a) genotypic polymorphisms of *FANCC* and *FANCD1*; (b) a combined effect of harboring a higher number of high-risk genotypes and smoking/passive smoking; (c) specific interactions of multiple genes, proteins encoded by which have been known to work jointly within the FA pathway. Our study permits a more precise and comprehensive evaluation of the lung adenocarcinoma risk associated with FA genes and a better insight into tumorigenesis of lung adenocarcinoma initiated by variant FA repair genes and how this is modified by exposure to cigarette smoke.

Though patients with FA are not more susceptible to lung cancer, somatic inactivation of the FA pathway has been observed in lung cancers [46]. Promoter hypermethylation of *FANCF* occurs in a significant proportion of lung cancer patients and is a significant predictor of poor survival [47]. This finding provides support for the biological plausibility of involvement of FA genes in lung cancer development. However, in considering whether our findings represent a true association between the SNPs of the FA genes and lung adenocarcinoma, the most important issue is the interpretation of the identified association between the SNPs and the trait. The present study used a candidate gene approach based on SNPs locating in the genes of the FA pathway. Because the SNPs analyzed do not affect amino acid coding and therefore probably do not directly affect protein function, the observed associations between cancer risk and SNPs should be interpreted as the presence of LD between these SNPs and other SNPs in exons (resulting in functional polymorphism) or in regulatory regions (affecting the expression of these genes). Furthermore, genetic heterogeneity is less of a concern in Taiwan than in the United States [31], and as a result, potential bias due to population stratification is less likely to be significant in our study, and the probability that the functional variants targeted by the same SNPs of individual FA genes are different in cases and controls due to differences in the genetic background of the two groups is small. However, we recognize that sequencing of the entire gene and promoter region is the definitive approach to identify all important sequence variants and that a large-scale evaluation of these variants and functional assessments are needed to address this question.

We previously proposed a hide-then-hit model [40], suggesting that the probability of manifesting the tumorigenic phenotype associated with low-penetrance alleles (such as SNPs) might depend on a joint effect between these polymorphic alleles and endogenous or exogenous risk factors. Our finding that tumorigenesis of lung adenocarcinoma due to FA genes was influenced by a cigarette smoke-related risk factor (Fig. 2) is consistent with this hypothesis. This finding suggests that, in subjects exposed to a greater cumulative amount of cigarette smoke, the lung cells are subjected to a larger amount of cross-linking agents and subsequently have a higher potential to develop lung adenocarcinoma if they have a suboptimal ability to maintain genomic stability because of harboring variant FA genes. The combined effect of FA genes and smoking-related risk factors leading to an increased risk of lung adenocarcinoma can also explain the tissue specificity if smoking or passive smoking creates a selective micro-environment beneficial for lung cells with abnormal FA function.

On the other hand, the hide-then-hit model [40], which suggests that a different range of disease phenotypes is caused by mutated forms or hypomorphic/polymorphic variants of the same genes, can also be applied to explain the lack of lung cancer predisposition in FA patients. Because the FA genes are essential, sub-optimal function due to mutated alleles of these genes would predispose cells to a high degree of genomic instability [39], leading to a severe decrease in proliferation and apoptosis caused by cell-cycle checkpoint, such as p53, thus reducing the likelihood that additional mutations will occur and allowing tumor formation. The importance of these findings is that escaping checkpoint surveillance is a critical element in the pathogenesis of cancer resulting from defective DNA repair mechanisms, such as the FA pathway, and it is probable that only mild phenotypic defects, such as slightly increased genomic instability resulting from suboptimal repair capacity associated with SNPs of FA genes, could meet this “hide-then-hit” requirement [40]. Our suggestion is in line with findings in cell culture and an animal model. Following DNA damage, embryonic fibroblasts from *Fancd2(−/−)* mice, but not *Fancd2(−/−)/p53(−/−)* mice, arrest [48]. Furthermore, loss of *p53* (i.e., heterozygosity for *p53*) significantly accelerated epithelial tumor formation in *Fancd2* knockout mice. Interestingly, when *Fancd2* mutant mice were crossed to mice with a null mutation in *p53*, lung adenocarcinomas were observed in these F*ancd2(−/−)/p53(+/−)* mice, but rarely in *p53(+/−)* mice, supporting the involvement of FA genes in lung cancer development.

The multigenic approach used in our study led to several joint effects within FA complexes being identified as significant in determining cancer risk. Recently, evidence has emerged for the cooperative involvement of different genes in disease etiology and cancer development. The combination of heterozygous abnormalities in different, but functionally related, genes is known to play a causal role in the pathogenesis of certain genetic syndromes [49]. Evidence for an increased level of genomic instability due to a combined effect of genes belonging to a common DNA repair pathway has been provided in a mouse model [50]. At the cellular level, the amount of DNA damage present in lymphoblastoid cell lines from healthy persons not exposed to a carcinogen is directly related to the number of variant alleles of genes involved in the DNA repair pathway [51]. Further support for a joint carcinogenic effect comes from observational studies. For instance, there is a trend to increased risk of developing breast cancer in women harboring a greater number of putative high-risk genotypes of major estrogen-metabolizing and DNA repair genes [52].

## Conclusion

In the present study, we addressed the issue of gene-gene interactions by examining joint effects among FA genes in relation to lung adenocarcinoma risk, but the novelty of our analysis is that it also tested these associations based on interactions known to operate among FA proteins at the molecular level. To the best of our knowledge, no epidemiological study has used this approach to examine gene-gene interactions in the FA pathway leading to cancer susceptibility. Certainly, given the number of comparisons and the sample size of the present study, the conclusions should be interpreted with caution and confirmed by other studies based on a larger sample size. However, our consistent identification of specific joint effects of FA genes within FA complexes leading to an increased risk of lung adenocarcinomas in both males and females provides support for a role of FA genes in determining lung adenocarcinoma susceptibility. Finally, the definition of FA genes in lung tumorigenesis is also of clinical importance. Platinum chemotherapeutic agents are used to treat a broad range of malignant diseases, including lung cancer, and alterations of DNA repair processes are known to be critical in mediating platinum resistance [53]. Because platinum forms DNA crosslinks to kill tumor cells, we are currently examining whether genetic polymorphism of FA genes identified as important in determining cancer risk is also associated with individual susceptibility to platinum chemotherapy in lung cancer treatment.

## Additional files

Additional file 1: Figure S1. Linkage disequilibrium (LD) block and estimates of the pairwise r^2^ for single-nucleotide polymorphisms (SNPs) of individual Fanconi anemia (FA) genes in Caucasians (CEU) and Asians (CHB + JPT, i.e., combination of Chinese and Japanese) from the HapMap release 22. The size of each triangle is proportional to the size of each FA gene in individual FA complexes. (PDF 113 kb) Additional file 2: Figure S2. Linkage disequilibrium (LD) between the single-nucleotide polymorphisms (SNPs) of individual Fanconi anemia (FA) genes genotyped in Taiwanese control men and women. The strength of the LD between SNPs, as indicated by the color scheme, was measured using a combination of the statistic D’ and the logarithm of the odds ratio for linkage (LOD) score (dark red shading, D’ = 1 and LOD score ≥ 2; light red shading, D’ < 1 and LOD score ≥ 2). The size of each triangle is proportional to the size of each FA gene in individual FA complexes. (PDF 109 kb) Additional file 3: Figure S3. Linkage disequilibrium (LD) between the single-nucleotide polymorphisms (SNPs) of *FANCC* genotyped in the cases and controls for both males and females in the present study. The strength of the LD between SNPs, as indicated by the color scheme, was measured using a combination of the statistic D’ and the logarithm of the odds ratio for linkage (LOD) score (dark red shading, D’ = 1 and LOD score ≥ 2; light red shading, D’ < 1 and LOD score ≥ 2). (PDF 110 kb)
