# Supplementary figures and images for: Fanconi anemia genes in lung adenocarcinoma- a pathway-wide study on cancer susceptibility
Source: J Biomed Sci. 2016 Feb 3;23:23. doi: 10.1186/s12929-016-0240-9 (PMC4739091; doi:10.1186/s12929-016-0240-9)

Figure S1.

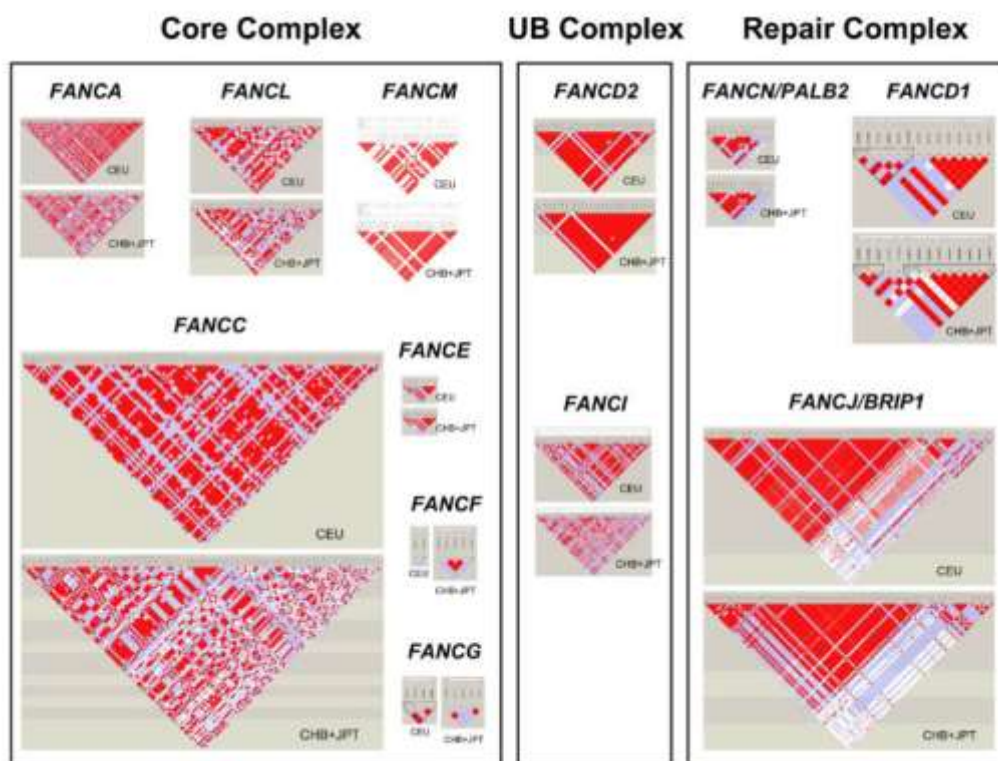

Supplement: Additional file 1: Figure S1. — Linkage disequilibrium (LD) block and estimates of the pairwise r2 for single-nucleotide polymorphisms (SNPs) of individual Fanconi anemia (FA) genes in Caucasians (CEU) and Asians (CHB + JPT, i.e., combination of Chinese and Japanese) from the HapMap release 22. The size of each triangle is proportional to the size of each FA gene in individual FA complexes. (PDF 113 kb) [file 12929_2016_240_MOESM1_ESM.pdf]

Supplementary Figure 2

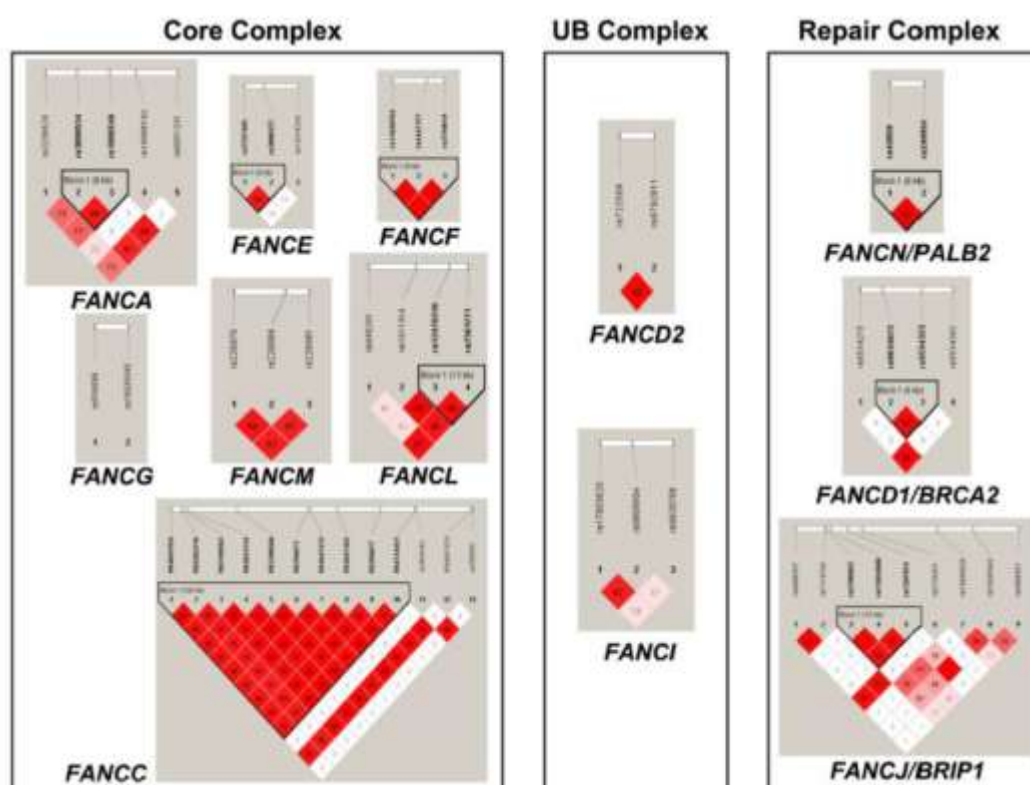

Supplement: Additional file 2: Figure S2. — Linkage disequilibrium (LD) between the single-nucleotide polymorphisms (SNPs) of individual Fanconi anemia (FA) genes genotyped in Taiwanese control men and women. The strength of the LD between SNPs, as indicated by the color scheme, was measured using a combination of the statistic D’ and the logarithm of the odds ratio for linkage (LOD) score (dark red shading, D’ = 1 and LOD score ≥ 2; light red shading, D’ < 1 and LOD score ≥ 2). The size of each triangle is proportional to the size of each FA gene in individual FA complexes. (PDF 109 kb) [file 12929_2016_240_MOESM2_ESM.pdf]

Supplementary Figure 3

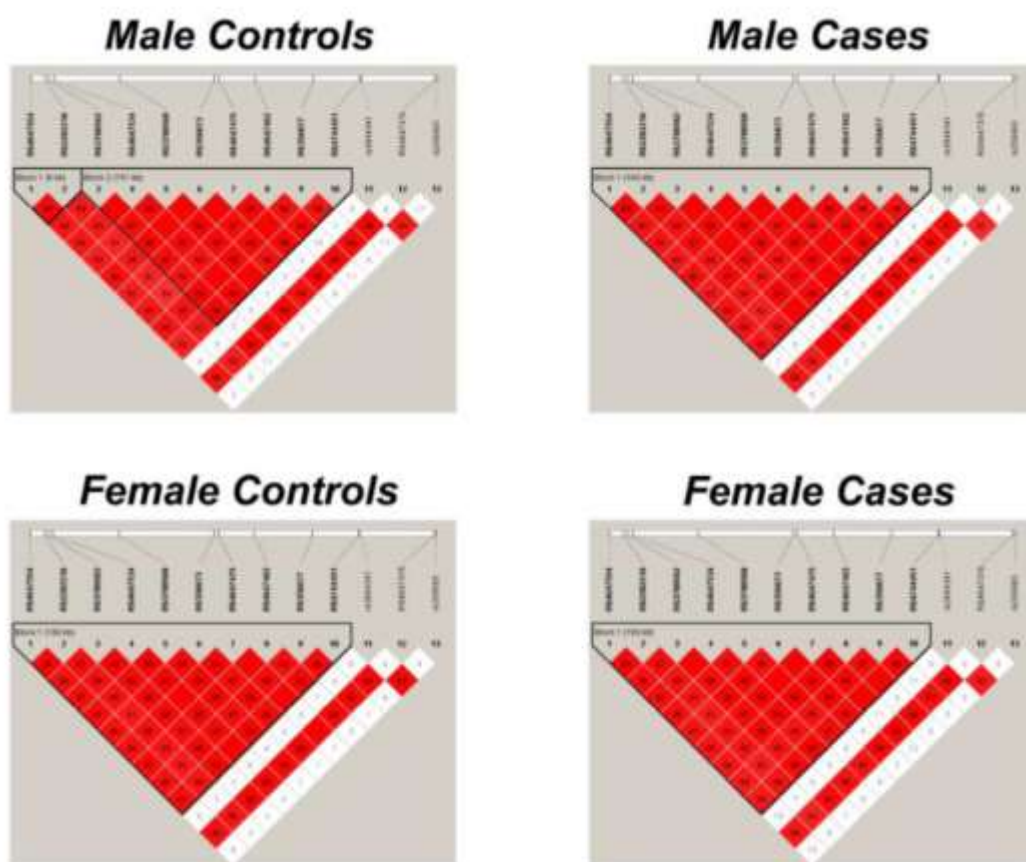

Supplement: Additional file 3: Figure S3. — Linkage disequilibrium (LD) between the single-nucleotide polymorphisms (SNPs) of FANCC genotyped in the cases and controls for both males and females in the present study. The strength of the LD between SNPs, as indicated by the color scheme, was measured using a combination of the statistic D’ and the logarithm of the odds ratio for linkage (LOD) score (dark red shading, D’ = 1 and LOD score ≥ 2; light red shading, D’ < 1 and LOD score ≥ 2). (PDF 110 kb) [file 12929_2016_240_MOESM3_ESM.pdf]
